# Illusory ownership of one’s younger face facilitates access to childhood episodic autobiographical memories

**DOI:** 10.1038/s41598-025-17963-6

**Published:** 2025-10-09

**Authors:** Utkarsh Gupta, Peter Bright, Alex Clarke, Waheeb Zafar, Pilar Recarte-Perez, Jane E. Aspell

**Affiliations:** 1 Department of Psychology, University of North Dakota, Grand Forks, ND USA; 2 School of Psychology, Sport and Sensory Sciences, Anglia Ruskin University, Cambridge, UK; 3 Department of Psychology, University of Cambridge, Cambridge, UK; 4 Department of Psychology, University of Warwick, Warwick, UK

**Keywords:** Bodily self-consciousness, Autobiographical memory, Enfacement illusion, Face ownership, Psychology, Human behaviour, Cognitive neuroscience

## Abstract

Our autobiographical memories reflect our personal experiences at specific times in our lives. All life events are experienced while we inhabit our body, raising the question of whether a representation of our bodily self is inherent in our memories. Here we explored this possibility by investigating if the retrieval of childhood autobiographical memories would be influenced by a body illusion that gives participants the experience of ownership for a ‘child version’ of their own face. 50 neurologically healthy adults were tested in an online enfacement illusion study. Feelings of ownership and agency for the face were greater during conditions with visuo-motor synchrony than asynchronous conditions. Critically, participants who enfaced (embodied) their child-like face recollected more childhood episodic memory details than those who enfaced their adult face. No effects on autobiographical semantic memory recollection were found. This finding indicates that there is an interaction between the bodily self and autobiographical memory, showing that temporary changes to the representation and experience of the bodily self impacts access to memory.

## Introduction

 When a person looks at their face in the mirror or looks down at their hands or legs, they usually experience an implicit sense of belongingness or ownership for these body parts^1–3^. The processing of body-related information and the generation of a representation of our body in our brain, independent of any conceptual or reflective processes, is termed bodily self-consciousness (BSC)^4–9^. The differentiation of one’s own body from the rest of the environment is based on the integration of multisensory bodily cues, and this gives rise to body ownership^2^. Body ownership (BO) is one of the key components of BSC. Other key components constituting BSC are the sense of agency (SoA), self-location (SL), and first-person perspective (FPP)^2,3,7,10,11^. SoA is the experience of having volitional control of one’s actions, FPP is the body-centred experience of a multimodal experiential space, and SL is the experience of where one’s self is located in space^3,12,13^. In neurologically healthy people, bodily self experiences including self location, agency and body ownership are associated with all personally-experienced life events^14^. If these bodily self experiences are encoded in memory, there must be interactions between bodily self representations and memory.

The conscious recollection of self-referenced personal experiences and factual knowledge is termed autobiographical memory (AM)^15^. AM can involve either a conscious reliving of a personal experience of a specific event from one’s past (EAM – episodic autobiographical memory) or recollection of factual knowledge about one’s own past or one’s self (SAM – personal semantic autobiographical memory)^16–18^. Since EAM concerns the conscious recollection of an event, it may include the re-experiencing of a younger version of one’s own body, as it was at the time of the original event. Theoretically, the ‘Self Memory System’ (SMS) model explores the underlying mechanism of the construction of AM^19^. The SMS model proposed the term ‘working self’ which refers to the interaction between self-representations and goal-driven working memory. Another key component of the SMS is the autobiographical knowledge base which includes episodic and factual autobiographical information. The SMS model holds that when cues such as visual, auditory, and other sensory stimuli are perceived, they trigger the interaction of the working self with an autobiographical knowledge base which facilitates the construction and recollection of AM^20^. It is possible that a temporary change in one’s body representation caused by an illusion of ownership for a body (face) part with features from a different time period in the past (i.e., child’s face) may trigger the activation of the childhood autobiographical knowledge base and facilitate enhanced recollection of an AM from that time period.

In previous studies, experimental full-body illusions (FBI) have been used to investigate the interaction between the bodily self and memory^5,21–27^. An FBI refers to a perceptual illusion in which a person experiences a distorted body representation for their entire body: it may include changes to which body one feels ownership for (i.e., participants may feel ownership for a mannequin/virtual body/another person’s body), their experience of where the self seems to be located may be altered, and there may also be changes to tactile representations^5,21–27^. Bergouignan and colleagues induced an illusory ‘out-of-body’ experience in participants wearing a virtual reality (VR) headset, through which they experienced and encoded an event from either the FPP (simulating an ‘in-body experience’) or the third-person perspective (TPP), simulating an ‘out-of-body experience’^21^. They assessed the impact of this illusory change in bodily self on memory recollection of an event by comparing the recollection performance between the ‘out-of-body experience’ and ‘in-body experience’ conditions. They found that recollection scores for emotional, factual, spatial, and temporal aspects of events encoded during the ‘out-of-body experience’ condition were significantly lower compared to the ‘in-body experience’ condition, suggesting an impairing effect of the illusion on memory encoding. In another FBI study by Iriye and Ehrsson^22^a sense of body ownership was induced for a mannequin viewed from the FPP in pre-recorded naturalistic 3D environments. Conditions of strong and weak sense of body ownership for the mannequin were created. Strong body ownership was created in synchronous conditions in which the participants saw touch on the mannequin and felt touch on their body at the same time and location. Weak body ownership was created in asynchronous conditions in which the participants saw touch on the mannequin and felt touch sensations on their body at different times and locations. The authors found that memory accuracy, immediate reliving, delayed emotional intensity, and vividness were enhanced for memories of the videos that were viewed while experiencing a strong sense of body ownership compared to weak sense of body ownership for the mannequin^22^.

In another FBI experiment, the impact of embodiment (i.e., the sense of being present in a body and having ownership and control over it) of a virtual avatar on memory recollection of navigation through a naturalistic virtual environment was assessed^14^. Stronger embodiment of the virtual avatar was induced by visuo-motor synchrony (i.e., the direction and time of movement of the virtual body matched with the movements of the participant’s body) compared with weaker embodiment (visuo-motor asynchrony) of the virtual avatar. The authors found that the weaker embodiment condition was associated with impaired incidental memory, perceptual details, contextual associations, and subjective sense of remembering^14^. The effects of a ‘body swap’ illusion on the encoding of episodic information has also been investigated^28^. While embodying their friend’s body via VR, participants rated their own personality traits. In a follow-up recognition memory task, it was found that there was impaired recognition of personality traits that were selected during the ‘body swap’ illusion^28^. This provided further evidence of impaired encoding of episodic information due to a change in body representation.

Two studies compared body, no-body and control (object) conditions in their effects on the encoding of everyday-life objects in an indoor or outdoor immersive virtual reality environment^29,30^. During the ‘body’ condition, the virtual environment was viewed by participants while viewing their own body from the FPP. In the ‘no-body’ condition the participants saw the virtual environment without their body being visually present in it, and in ‘control’ conditions a non-humanoid virtual object was seen in the environment instead of a body. A recognition task was used to assess the ability of participants to recognise and recollect objects (such as coffee machine, pen, and trash bin) viewed in the virtual environment. The control condition was added to the research design to investigate if the possible differences in object recognition performance between conditions are due to vision and attention distractors or if the differences can be explained by the effect of body embodiment alone. It was found that the ‘body’ condition was associated with better performance during the recognition task than the ‘no-body’ and ‘control’ conditions^29,30^. These findings suggest that the mere presence of bodily cues facilitates the encoding of object information of an episodic event.

Previous research has therefore provided evidence of an interaction between the bodily self and the encoding and retrieval of new memories, but whether and how previously encoded AMs might be impacted by manipulations of the bodily self has not been explored to date. Studies have shown that the neural mechanisms of recollecting episodic details in a laboratory based episodic memory task (when participants are required to e.g. retrieve memories of encoded words or pictures) are different from those underlying the recollection of autobiographical episodic details (during e.g., an autobiographical memory interview which requires participants to recollect details of events from earlier time periods)^31–33^. In a study by Chen and colleagues, participants were instructed to study a set of scenes, after which they undertook two types of memory tasks. A picture memory task – requiring participants to report if a picture was from the studied set of scenes - was used to test episodic memory recollection. To test autobiographical memory recollection, participants were asked to report (via a yes/no response) if the presented scenes reminded them of an event from a pre-experimental time period. The authors found that the autobiographical recollection task activated the default mode network, whereas the episodic picture memory task activated the parietal and frontoparietal brain regions^31^. Further, in a review by Gilboa, it was argued that existing studies on laboratory based episodic memory tasks showed activation in different brain areas compared to those found during autobiographical memory recollection^32^. Right mid-dorsolateral prefrontal cortex activation was more associated with episodic memory retrieval than autobiographical recollection. Further, ventromedial prefrontal cortex activation was more associated with autobiographical recollection than episodic memory retrieval^32^. It has also been previously shown that medial prefrontal cortex mediates the activation of one’s self representation during encoding of a memory which influences autobiographical recollection and its veracity^32^. Compared to self-referential processing during autobiographical memory recollection, laboratory based episodic memory retrieval depends more on monitoring of omission, commissions, and repetitions^32,33^. Since autobiographical memories and laboratory based episodic memories are arguably different processes with different neural correlates, their interaction with bodily self may arguably differ as well. However, previous literature investigates and provides evidence only for an interaction between bodily self and laboratory based episodic memory^14,21,22,28–30^.Therefore, it is important to investigate how alterations in the bodily self may affect AM for events from pre-experiment time periods. One well-studied way to manipulate the bodily self is by inducing a change in face ownership via the enfacement illusion^34,35^. Enfacement refers to the sense of having ownership and control over a face. Previous studies have employed either congruent visuo-tactile cues (where the viewed face is touched at the same time and same place as one’s own) or congruent visuo-motor cues (where the viewed face moves at the same time and in the same way as one’s own) to generate an enfacement illusion^34,36^.

The present study implemented an online version of the visuo-motor enfacement illusion to generate ownership for either a morphed ‘child version’ of the participant’s own face or their own unmorphed face viewed in a live video on a computer screen. Visuo-motor stimulation was selected over visuo-tactile stimulation for the enfacement illusion as it was a more feasible option for our online experimental protocol, it was more naturalistic, and provided a strong sense of embodiment and agency (volitional control over movements) by directly linking visual feedback and motor movements. The effect of the enfacement illusion on the recollection of both childhood and recent autobiographical memories was measured. We hypothesised that (i) recollection of episodic details of childhood memories would be enhanced after participants enfaced a child version of their own face compared to after they enfaced the unmorphed version, (ii) the effect would be greater after the synchronous (strong illusion) condition compared to asynchronous (weak illusion) condition. In order to test for the specificity of any effects of the illusion on childhood autobiographical recollection, we included control conditions in which we measured recollection of recently experienced episodic and personal-semantic memories. We did not expect recollection of these recent details be influenced by the illusion.

## Results

### Enfacement illusion

A 2 × 2 × 5 mixed model multivariate analysis of variance (MANOVA) was conducted to examine the effect of a between-subject factor of group (child face filter, no face filter) and a within-subject factor of visuo-motor synchrony (synchronous, asynchronous) on enfacement scores (questions pertaining to face ownership, face agency, child-like experience, adult-like experience, and a control question). Assumptions of multivariate normality, presence of outliers, homogeneity of covariance matrices and absence of multicollinearity for MANOVA were checked. Three multivariate outliers were identified using the Mahalanobis distance with values greater than the critical value of 20.52 for 5 degrees of freedom. The outliers were excluded from further analyses. The means and standard deviations for enfacement scores are provided in Table 1. Each subgroup had a sample size greater than 20 and the distribution was considered normal in agreement with central limit theorem. Mauchly’s Test of Sphericity indicated that the assumption of sphericity was violated for enfacement scores, X^2^(9) = 6.121, *p* <.001, therefore degrees of freedom were corrected using Greenhouse-Geisser estimates of sphericity (ε = 0.583). There were no correlation coefficients between enfacement scores greater than 0.80 suggesting absence of multicollinearity^37^.

**Table 1:** Descriptive statistics showing means and standard deviations of enfacement illusion scores (face ownership, face agency, child-like experience, adult-like experience, and control scores) for the factors group (child face filter, no face filter) and visuo-motor synchrony (synchronous, asynchronous).

	Synchrony	Group	Mean	SD	*N*
**Face ownership**	Sync	Child face filter	4.81	1.26	50
		No face filter	5.12	1.35	49
	Async	Child face filter	3.95	1.37	49
		No face filter	4.73	1.34	49
**Face agency**	Sync	Child face filter	5.65	1.00	50
		No face filter	5.78	1.17	49
	Async	Child face filter	5.04	1.26	49
		No face filter	5.73	1.05	49
**Child-like experience**	Sync	Child face filter	4.65	1.60	50
		No face filter	2.33	1.47	49
	Async	Child face filter	4.38	1.75	49
		No face filter	2.36	1.38	49
**Adult-like experience**	Sync	Child face filter	3.73	1.57	50
		No face filter	5.52	1.23	49
	Async	Child face filter	3.83	1.72	49
		No face filter	5.51	1.46	49
**Control scores**	Sync	Child face filter	3.14	1.71	50
		No face filter	2.39	1.57	49
	Async	Child face filter	3.33	1.71	49
		No face filter	2.57	1.78	49

Note. Maximum score for subjective experience of illusion was 6; SD – Standard deviation; N – Sample size for each condition.

**Fig. 1:**
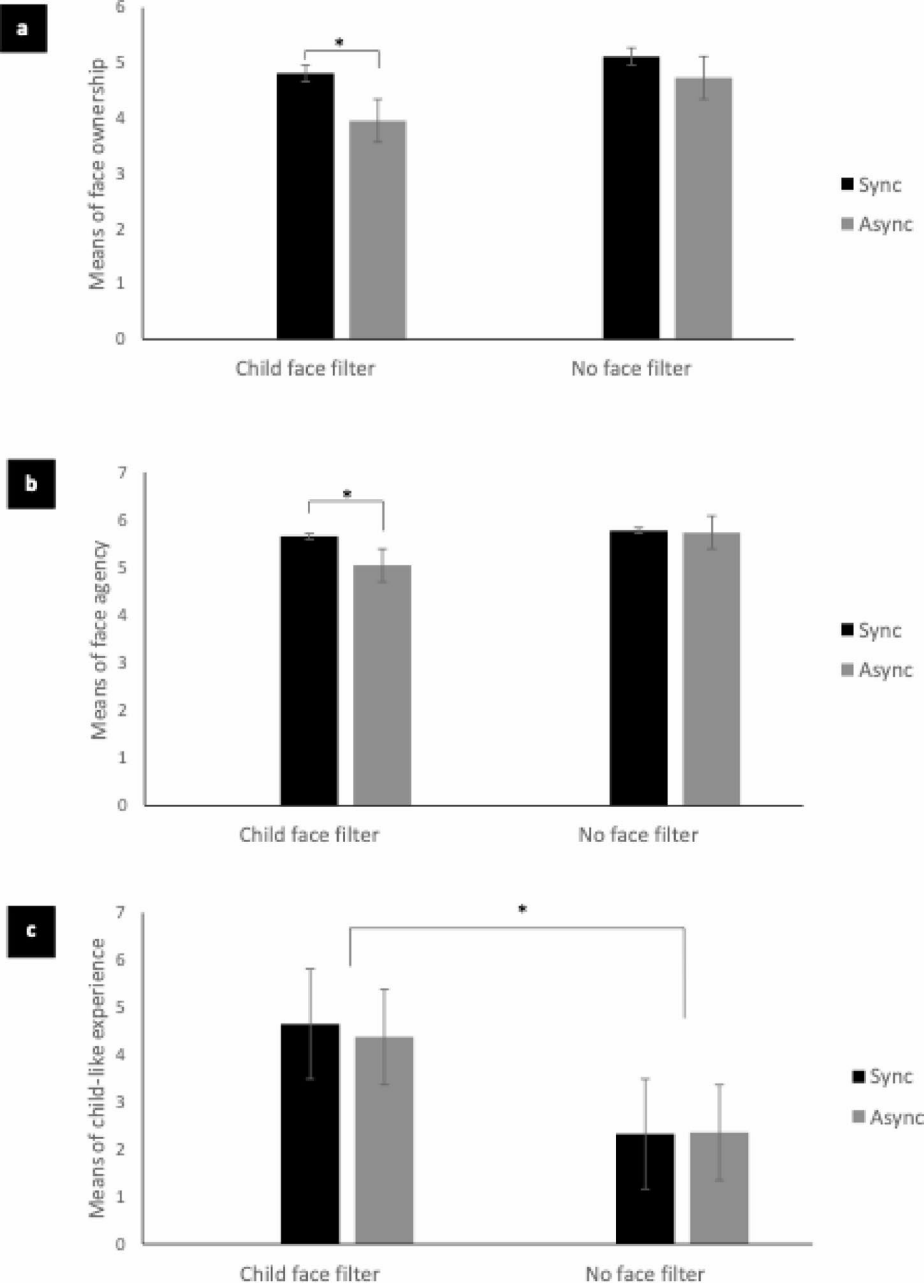
*Histograms showing differences in enfacement illusion scores based on group (child face filter*,* no face filter) and visuo-motor synchrony (synchronous*,* asynchronous).* *Note.* **(a)** A histogram showing differences in face ownership scores based on group (child face filter, no face filter) and visuo-motor synchrony (synchronous, asynchronous); **(b)** A histogram showing differences in face agency scores based on group (child face filter, no face filter) and visuo-motor synchrony (synchronous, asynchronous); **(c)** A histogram showing differences in child-like experience scores based on group (child face filter, no face filter) and visuo-motor synchrony (synchronous, asynchronous). Error bar indicates standard error. A star (*) indicates a significant difference at *p* value less than 0.05.

Results from the mixed model MANOVA showed that there was a significant within-subject effect of visuo-motor synchrony on enfacement scores, (*F*(1,48) = 5.273, *p* =.026). However, there was no significant interaction between visuo-motor synchrony and group effects, (*F*(1,48) = 1.902, *p* =.174). The results showed that enfacement scores significantly differed within subjects (*F*(2.334,112.022) = 40.746, *p* <.001). There was a significant interaction effect between enfacement illusion scores and group (*F*(2.334,112.022) = 18.996, *p* <.001) and visuo-motor synchrony (*F*(3.407,163.555) = 2.770, *p* =.018). Pairwise comparisons showed that face ownership (*p* =.006) and face agency (*p* =.005) scores were significantly greater following the visuo-motor synchronous condition compared to the asynchronous condition for participants in the child face filter group (Fig. 1a and b). However, there were no significant differences in face ownership (*p* =.202) and face agency (*p* =.465) scores between synchronous and asynchronous conditions for participants in the no face filter group. Further, there were no significant differences (*p* >.05) in child-like experience, adult-like experience, or control scores based on visuo-motor synchrony. Pairwise comparison also showed that participants in the child face filter group had significantly greater child-like experience scores (*p* <.001, Fig. 1c) compared to participants in the no face filter group. Further, participants in the child face filter group had significantly lower adult-like experience scores (*p* <.001) compared to no face filter group. Face ownership, face agency, and control scores did not significantly (*p* >.05) differ between groups.

## Interrater reliability test for AMI scoring

The intraclass correlation coefficient (ICC) test was performed to assess the reliability of the scoring of AMI undertaken by multiple researchers. Memory interviews from 10 randomly selected participants were scored separately by UG and PRP. The raters were blind to the group distribution of the participants. Results for the first attempt of interrater reliability between the two raters’ scoring showed poor to moderate reliability for memory scores based on semantic questions (*ICC* = 0.427; 95% CI [0.031, 0.716]), free recollection (*ICC* = 0.184; 95% CI [−0.195, 0.491]), and specific probing (*ICC* = 0.377; 95% CI [−0.211, 0.718]). The scores were reassessed and discussed to come to similar scoring approach. The memories from second set of randomly selected 10 participants were then rescored separately by UG and PRP. The revised ICC test results for interrater reliability showed good to excellent reliability for memory scores based on semantic questions (*ICC* = 0.899; 95% CI [0.810, 0.946]), free recollection (*ICC* = 0.966; 95% CI [0.932, 0.982]), and specific probing (*ICC* = 0.916; 95% CI [0.251, 0.975]). We achieved greater agreement for free recall and specific probing based composite scores compared to the original work done by Levine and colleagues where reliability was tested among four scorers^38^. They achieved an ICC value of 0.79 for free recall and 0.41 for specific probing based memory scores. The authors did not report corresponding confidence intervals for their ICC values. The authors argued that the lower ICC value for specific probing was due to limited range of scores as most participants received maximum scores. Arguably, the limited range in specific probing based memory scores can also explain the lower confidence in our inter-rater reliability. After achieving good to excellent interrater reliability, the memories from the remaining 40 participants (20 each by UG and PRP) were scored.

## Effect of child face filter on childhood and recent AM performance

Out of a total of 200 memories that were obtained from 50 participants using the AMIs, 100 (2 per participant) were childhood autobiographical memories (CAMs) and 100 (2 per participant) were recent autobiographical memories (RAMs). A 2 × 2 × 2 × 3 mixed model MANCOVA was performed to analyse the effect of group (child face filter, no face filter), visuo-motor synchrony (synchronous, asynchronous), and AM life period type (recent, childhood) on AM recollection scores (i. semantic, ii. free recollection, and iii. specific probing based memory scores) with age as a covariate. Assumption of linearity for age as a covariate was met as linear patterns were observed in scatterplots between age and autobiographical memory scores grouped for each independent variable condition. The Box’s M of 133.475 indicates that the homogeneity of covariance matrices across groups is assumed (*F*(78,7275.648) = 1.250, *p* =.068). Assumption of homogeneity of regression slopes for age as covariate was met as there were no significant interaction of age with visuo-motor synchrony (Wilk’s lambda = 980, *F*(1,47) = 0.941, *p* =.337, pη^2^ = 0.020), and life period of recollected autobiographical memory (Wilk’s lambda = 980, *F*(1,47) = 0.941, *p* =.337, pη^2^ = 0.020). A Mann-Whitney U test showed that age did not significantly differ between the two groups (*U* = 286.00, *p* =.605). Assumptions of multivariate normality, presence of univariate and multivariate outliers, homogeneity of covariance matrices and absence of multicollinearity for MANCOVA were checked. Two univariate outliers were identified in the child face filter group for recollection of semantic childhood AM following synchronous visuo-motor stimulation and for recollection of semantic recent AM following asynchronous visuo-motor stimulation. However, the data points were legitimate and not excluded from further analyses. No multivariate outliers were identified using the Mahalanobis distance as no values were above the critical value of 16.27. The analysis was assumed to be robust against the violation of normality in agreement with the central limit theorem, as the size of the sample in each condition was more than 20. Mauchly’s Test of Sphericity indicated that the assumption of sphericity was violated for AM recollection scores, X^2^(2) = 6.664, *p* =.036, therefore degrees of freedom were corrected using Huynh-Feldt correction (ε = 0.952) as Greenhouse-Geisser epsilon value (ε = 881) was greater than 0.75. The dependent variables showed weak to moderate correlation with no correlation coefficient greater than 0.80, suggestive of absence of multicollinearity^37^. The descriptive statistics for childhood and recent AMs are presented in Table 2.

**Table 2:** Descriptive statistics showing means and standard deviations of childhood and recent autobiographical memory scores (composite semantic scores, composite free recollection scores, composite specific probing scores) for the factors group (child face filter, no face filter) and visuo-motor synchrony (synchronous, asynchronous).

Life period	Memory details	Group	Synchrony	Mean	SD	*N*
**Childhood**	**Composite-semantic**	Child face filter	Sync	0.926	0.128	25
			Async	0.914	0.136	25
		No filter	Sync	0.926	0.118	25
			Async	0.913	0.119	25
	**Composite-FR**	Child face filter	Sync	1.919	0.534	25
			Async	1.934	0.531	25
		No filter	Sync	1.560	0.554	25
			Async	1.513	0.614	25
	**Composite-SP**	Child face filter	Sync	2.413	0.327	25
			Async	2.340	0.413	25
		No filter	Sync	2.134	0.459	25
			Async	2.060	0.531	25
**Recent**	**Composite-semantic**	Child face filter	Sync	0.926	0.109	25
			Async	0.893	0.166	25
		No filter	Sync	0.906	0.119	25
			Async	0.959	0.074	25
	**Composite-FR**	Child face filter	Sync	1.648	0.464	25
			Async	1.688	0.544	25
		No filter	Sync	1.426	0.547	25
			Async	1.674	0.648	25
	**Composite-SP**	Child face filter	Sync	2.474	0.283	25
			Async	2.473	0.346	25
		No filter	Sync	2.314	0.477	25
			Async	2.333	0.581	25

Note. Maximum score for memory was 3; SD – Standard deviation; N – Sample size for each condition; Composite FR – Composite free recollection scores; Composite SP – Composite specific probing scores.

**Fig. 2:**
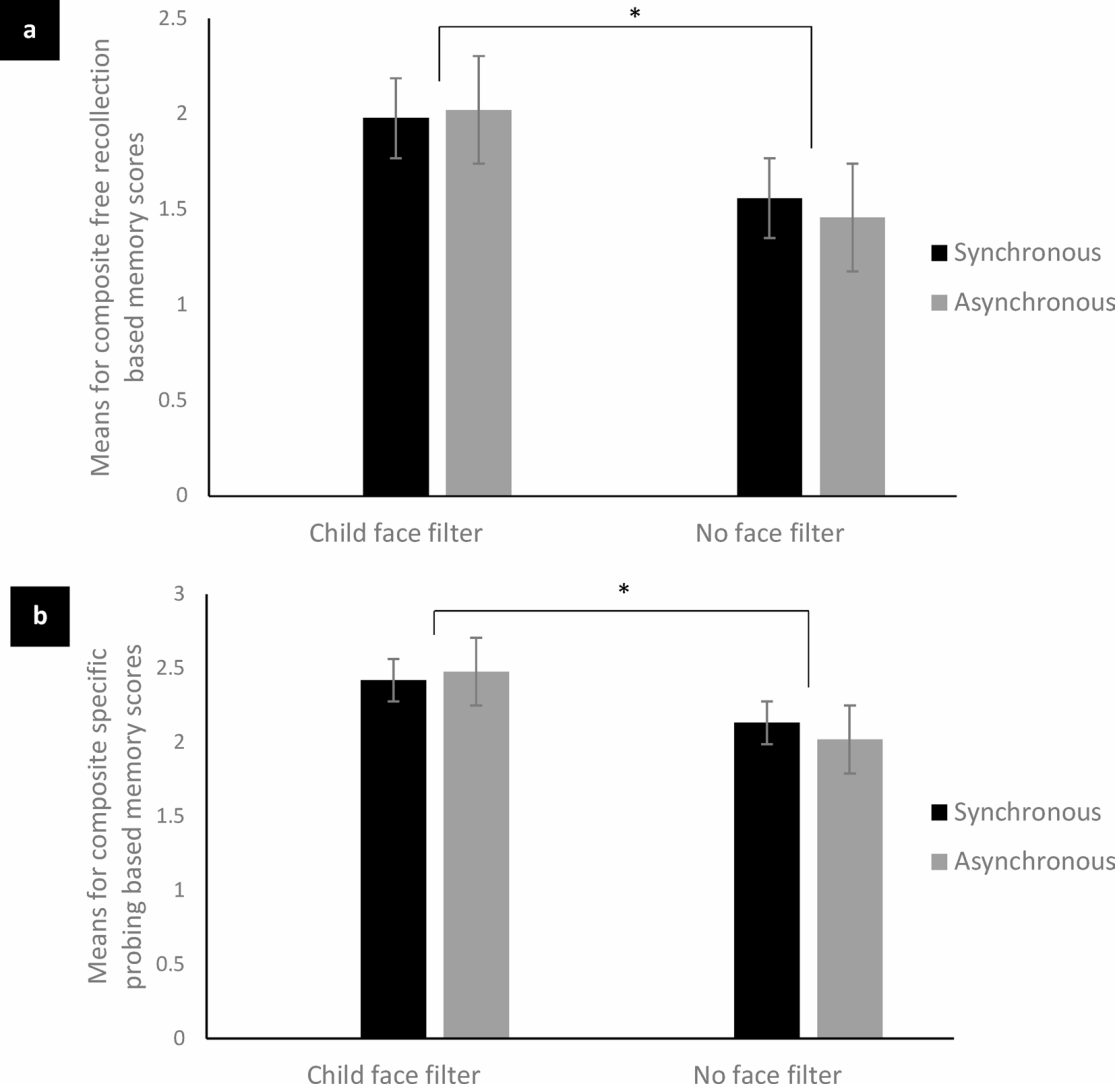
*Histograms showing differences in childhood AM scores for factors group (child face filter*,* no face filter) and visuo-motor synchrony (synchronous*,* asynchronous).* *Note*. **a**. A histogram showing differences in composite free recollection based childhood AM scores for factors group (child face filter, no face filter) and visuo-motor synchrony (synchronous, asynchronous); **b**. A histogram showing differences in composite specific probing based childhood AM scores for factors group (child face filter, no face filter) and visuo-motor synchrony (synchronous, asynchronous). Error bar indicates standard error. A star (*) indicates a significant difference at *p* value less than 0.05.

Results from the mixed model MANCOVA showed that age did not significantly interact with AM recollection scores (Wilk’s lambda = 884, *F*(2,46) = 3.024, *p* =.059, pη^2^ = 0.116) indicating that its adjustment was not required. There was a significant within subject effects on AM recollection scores (*F*(1.904,89.470) = 14.941, *p* <.001). There was significant interaction effect of group (*F*(1.904,88.022) = 4.457, *p* =.014) on AM recollection scores. However, we did not find any significant effect of visuo-motor synchrony on AM recollection scores (*F*(2,94) = 0.714, *p* =.492). No other significant interactions among group, AM life period, visuo-motor synchrony, and AM recollection scores were observed. Pairwise comparisons with Bonferroni adjustment for multiple comparisons showed that for CAM recollection following visuo-motor synchronous stimulation, both episodic free recollection (*p* =.024) and specific probing based recollection (*p* =.018) scores were significantly greater for participants in the child face filter group compared to participants in the no face filter group (Fig. 2a and b). The findings were similar for CAM recollection following visuo-motor asynchronous stimulation and both episodic free recollection (*p* =.014) and specific probing based recollection (*p* =.015) scores were significantly greater for participants in the child face filter group compared to participants in the no face filter group. However, there were no significant differences in semantic CAM recollection scores (*p* >.05) between the two groups following both visuo-motor synchronous and asynchronous stimulation. No significant differences (*p* >.05) were observed in semantic, episodic free recollection, and specific probing based RAM recollection scores between the two groups. Further pairwise comparison showed that the participants in the child face filter group recollected significantly more free recollection based episodic details (*p* =.005) of CAM following visuo-motor synchrony compared to RAM, whereas the participants in the no face filter group recollected significantly more specific probing based episodic details (*p* =.020) of RAM following visuo-motor asynchrony compared to CAM. The semantic, free recollection, and specific probing based CAM and RAM recollection scores did not significantly differ (*p* >.50) between visuo-motor synchronous and asynchronous conditions.

## Correlations between enfacement illusion scores and childhood autobiographical memory scores

Before analysing the correlation between the enfacement score (face ownership, face agency) and childhood autobiographical memories scores (i. semantic, ii. free recollection, and iii. specific probing based memory scores), the scores were first checked for outliers and normal distribution. There was a non-normal distribution of composite memory scores for semantic memory (*n* = 100, *W* = 0.666, *p* <.001), free recollection based episodic memory (*n* = 100, *W* = 0.973, *p* =.037), and specific probing based episodic memory scores (*n* = 100, *W* = 0.959, *p* =.003). A non-normal distribution was also found for face agency (*n* = 100, *W* = 0.927, *p* <.001). A series of non-parametric Spearman correlations were performed.

There were no significant correlations between childhood autobiographical memory scores and enfacement scores in participants belonging to ‘child face filter’ group (Table 3). However, in the participants belonging to the ‘no face filter’ group, face agency scores showed a significant positive correlation with memory scores based on specific probing (*r*(23) = 0.627, *p* <.001).

**Table 3:** *Correlations between autobiographical memory scores (composite semantic scores*,* composite free recollection scores*,* composite specific probing scores) and enfacement scores (face ownership*,* face agency).*

Life period	Group	Synchrony	AM scores	FO	FA
**Childhood AM**	**Child face filter**	**Sync**	**S**	− 0.075 (*p* =.723)	− 0.338 (*p* =.099)
			**FR**	0.040 (*p* =.849)	0.039 (*p* =.854)
			**SP**	0.159 (*p* =.448)	0.094 (*p* =.448)
		**Async**	**S**	− 0.137 (*p* =.514)	− 0.235 (*p* =.259)
			**FR**	0.019 (*p* =.929)	− 0.166 (*p* =.429)
			**SP**	− 0.076 (*p* =.718)	− 0.218 (*p* =.296)
	**No filter**	**Sync**	**S**	− 0.076 (*p* =.717)	0.062 (*p* =.769)
			**FR**	0.229 (*p* =.271)	0.336 (*p* =.101)
			**SP**	0.441 (*p* =.027)	0.280 (*p* =.175)
		**Async**	**S**	0.463 (*p* =.020)	0.352 (*p* =.085)
			**FR**	0.547 (*p* =.005)	0.464 (*p* =.019)
			**SP**	0.429 (*p* =.032)	0.627 (*p* <.001)*
**Recent AM**	**Child face filter**	**Sync**	**S**	0.208 (*p* =.318)	− 0.138 (*p* =.511)
			**FR**	− 0.176 (*p* =.399)	− 0.119 (*p* =.572)
			**SP**	0.155 (*p* =.459)	0.090 (*p* =.670)
		**Async**	**S**	− 0.036 (*p* =.864)	0.027 (*p* =.899)
			**FR**	0.228 (*p* =.273)	0.146 (*p* =.488)
			**SP**	0.051 (*p* =.807)	0.294 (*p* =.153)
	**No filter**	**Sync**	**S**	− 0.077 (*p* =.716)	− 0.100 (*p* =.635)
			**FR**	0.280 (*p* =.175)	0.286 (*p* =.166)
			**SP**	0.188 (*p* =.368)	0.272 (*p* =.189)
		**Async**	**S**	− 0.104 (*p* =.619)	− 0.085 (*p* =.686)
			**FR**	− 0.046 (*p* =.828)	0.122 (*p* =.562)
			**SP**	− 0.043 (*p* =.837)	0.034 (*p* =.870)

Note. AM –Autobiographical memories; S – Composite semantic memory scores; FR – Composite free recollection scores; SP – Composite specific probing scores; FO – Face ownership; FA – Face agency; Sync – visuomotor synchrony; Async – visuomotor asynchrony. A star (*) indicates an adjusted (Holm-Bonferroni correction) significant spearman’s correlation at *p* value less than 0.001.

## Correlations between enfacement illusion scores and recent autobiographical memory scores

Before analysing the correlation between the enfacement scores (face ownership, face agency) and recent autobiographical memories scores (i. semantic, ii. free recollection, and iii. specific probing based memory scores), the scores were first checked for outliers and normal distribution. There was non-normal distribution of composite memory scores for semantic memory (*n* = 100, *W* = 0.660, *p* <.001) and specific probing based episodic memory scores (*n* = 100, *W* = 0.931, *p* <.001). Non-normal distribution was also found for face agency (*n* = 100, *W* = 0.925, *p* <.001). A series of non-parametric Spearman correlations were performed. However, no significant correlations were found between recent autobiographical memory scores and enfacement scores (Table 3).

## Discussion

In the present study, we hypothesised that enfacing a younger version of one’s own face would facilitate better recollection of episodic details of childhood AMs, compared to enfacement of one’s own ‘unfiltered’ face viewed on a screen. In keeping with our hypothesis, we found that participants who enfaced a child-like version of their own face demonstrated a more detailed recollection of episodic childhood AMs than participants who enfaced their unaltered face. Arguably, providing the relevant bodily triggers or cues, via an enfacement illusion may therefore facilitate the recollection of greater episodic details of an event experienced when the participant was younger. Further pairwise comparison showed that participants in the child face filter group recollected greater episodic details during free recollection of childhood AMs compared to recent AMs following visuo-motor synchronous condition. In accord with the hypothesis, it can also be predicted that participants in the no face filter group would have been able to recollect greater details of recent AMs compared to participants in the child face filer group. We found that recent AMs did not significantly differ between ‘child face filter’ and ‘no face filter’ groups. However, pairwise comparisons showed that participants in the no face filter group recollected more episodic details during specific probing based recollection of recent AMs compared to childhood AMs. Interestingly, this effect was found in the visuo-motor asynchronous condition and not in the synchronous condition. It can be speculated that while viewing one’s unaltered face in the asynchronous condition, there is diverted attention on recollection of AMs rather than experiencing the visuo-motor conflict. In support of this speculation, this difference was observed only for specific probing based recollection requiring more attention to details than free recollection. Nevertheless, these findings are the first to suggest that temporary changes to the representation of one’s body may modulate access to (and guided recollection of) autobiographical memory.

In the current study we also predicted that visuo-motor synchrony would generate a stronger illusion of enfacement of the participant’s younger face compared to visuo-motor asynchrony. We hypothesised that generating a stronger illusion of enfacement for a participant’s younger face would facilitate the recollection of childhood AM details. Results from the present study show that we were able to generate a significantly stronger illusion of enfacement of a younger version of the participants’ own faces by implementing visuo-motor synchrony compared to asynchrony. However, contrary to our expectation, visuo-motor synchrony did not significantly affect childhood nor recent AM recollection. We found no evidence that responses to the illusion questionnaire items about feeling younger/older were modulated by visuo-motor synchrony. The lack of synchrony effect on memory recollection is an important limitation of the present study. However, the lack of synchrony effect may be because the head movements still had temporal synchrony during the asynchronous condition: i.e., when the participant moved their head to the right, the face on the screen moved to the left at exactly the same time. Additionally, evidence from previous literature shows that visual dominance alone can induce an illusion. Maselli and Slater^39^ and Aizu and colleagues^40^ found that a body ownership illusion can be induced by viewing a virtual body without the need of additional congruent sensory or motor stimulation. Therefore, the conditions of visuomotor synchrony and asynchrony were perhaps not sufficiently different to drive differences in memory recollection or modulate the experience of having a younger face.

We recommend future enfacement illusion studies attempt to generate a stronger illusion of ownership for one’s younger face via a lab-based study and possibly also testing the visuo-tactile version of the enfacement illusion. Given that our findings showed no effect of synchrony on memory recollection, it is important to investigate if eliciting a stronger enfacement illusion may have an effect on AM recollection that is synchrony-specific. This would be stronger evidence that the effect of the experimental setup on memory recollection is due to a change in embodiment (enfacement), rather than a less-specific priming effect. The current enfacement illusion had limited strength as the Snapcamera app did not allow us to accurately control for age and resemblance of the morphed childlike faces to the participants’ faces when they were children. This limitation could be overcome by using ‘deep fake’ AI software in future experiments. Another limitation of the current research procedure was having the enfacement questionnaire being completed by the participants immediately after the illusion protocol and before the AM interview. This could have also created an expectancy effect, potentially biasing participants’ responses by alerting them to the objectives of the study. To overcome this limitation, we recommend a modified protocol for future research where a second session of the illusion protocol is carried out after the AM interview and the enfacement questionnaire is instructed to be completed only after the repeated illusion protocol. We also recommend it be run in a lab rather than online.

However, the lack of visuo-motor synchrony effect on AM recollection may also indicate that the significant difference in AM recollection between child face filter group and no face filter group may be due to the priming effect of viewing a childlike face, i.e. it unconsciously activates memories of one’s childhood^41,42^. Priming has previously been categorised as autobiographical-source priming or semantic-source priming^41^. An autobiographical source refers to a personally-experienced episodic stimulus such as a memory of a trip to a specific place or reminiscence about another specific event from one’s past, whereas a semantic source refers to personally meaningful words or pictures. It has been argued that priming from semantic sources may also activate autobiographical memories^43^. For example, when we see the word ‘garden’, this will not only prime semantically-related items but will also prime autobiographical memories related to gardens. In this example, the source of the priming is semantic in nature and this is termed semantic-to-autobiographical priming^43^. If viewing a childlike face is considered as a semantic source of priming, one might expect it to affect recollection of both personal-semantic and episodic AM details. However, in the current study we found that the facilitation of childhood AM by viewing a child-like version of one’s own face was specific to the recollection of episodic childhood AM details and was not found for personal-semantic childhood AM details. This suggests that the effect of our enfacement illusion on memory retrieval is not only due to priming but may alternatively (or also) be due to the effects of enfacing (embodying) the childlike face. In order to investigate this further, future studies should directly compare a condition of simple priming (e.g. showing a picture of a childlike face) with the enfacement illusion to investigate whether the effect of an enfacement illusion on episodic AM recollection is greater than effects due to priming.

Theoretically, a possible explanation of the current findings is that enfacement of a childlike face facilitated the recollection of greater episodic details of an event experienced when the participant was younger irrespective of strong (visuo-motor synchronous condition) or weak (visuo-motor asynchronous condition) conditions of the enfacement illusion. It can be argued that encoded episodic details of a particular event includes the bodily information registered by the brain during that event. The bodily information available during any given event may constitute one of the key components of the memory traces that contribute to the construction of a memory, as proposed in Multiple Trace Theory (MTT) and Trace Transformation Theory (TTT)^44,45^. This argument is supported by the results of a full body illusion study by Iriye, Chancel and Ehrsson^46^. Participants were viewed naturalistic 3D pre-recoded videos in which they viewed a mannequin from the FPP. Simultaneous functional magnetic resonance imaging (fMRI) was performed. Participants were instructed to recollect memories of the video one week later when a second fMRI scan took place. The authors found that activation of brain areas associated with memory trace formation - i.e., right hippocampus and angular gyrus^44^ - was significantly different for events encoded during a strong body ownership condition compared to a weak body ownership condition^46^. The authors further found that activity patterns in the left hippocampus during memory encoding were significantly reinstated during the recollection of memories associated with stronger body ownership compared to weaker body ownership^46^. A study by Meyer and colleagues also found links between hippocampal activity and the bodily self: hippocampal reinstatement was stronger (and coupled with premotor cortex reinstatement) when the sense of agency was preserved during in a VR setting during fMRI scanning^47^. These recent findings suggest there is an interaction of bodily self and memory representations at the level of memory trace formation and thus support our argument.

The Self Memory System (SMS) model was proposed to explain the link between AM and self^19,48^. The SMS model incorporates the claim that the conceptual self interacts with an episodic memory system. The episodic memory system includes event-specific knowledge related to autonoetic experience of mentally reliving the past. However, how the bodily self may contribute to the construction of AMs has not previously been explored. Since we were able to show that embodying a child-like version of one’s own face may have facilitated detailed recollection of childhood memories, we suggest that memory traces constructed from bodily inputs present at the time of encoding contribute to the episodic memory system of the SMS model, and eventually to the construction of AMs. We therefore argue that by triggering the memory traces with childlike bodily cues may facilitate the recollection of episodic details that were previously inaccessible. A recent study by Yates and colleagues on infants showed that there was greater hippocampal activity when infants viewed previously unseen photographs in a memory task suggesting that there was a behavioural effort to search for previously encoded memories^49^. The authors suggest that it is possible that memory formation occurs as early as 1 year of age and impaired recollection of early age memories indicate inability to access these memories^49^. In keeping with the suggestion, our finding of facilitated recollection of childhood autobiographical memories following enfacement of a childlike face arguably indicates that there were episodic details that were previously inaccessible. Further, our findings create future research opportunities where manipulation of the implicit bodily self can be explored to access previously inaccessible autobiographical memories such as the memories from childhood amnesia phase.

Unexpectedly, we also observed a positive correlation between episodic childhood AM recollection scores and enfacement (face agency) scores during the condition without any face filter and following asynchronous visuo-motor stimulation. Such an association was not observed for conditions in which child face filter was applied. A possible explanation for this finding could be linked to challenges in self-coherence during incongruent visuo-motor stimulation while viewing one’s own face. Asynchronous stimulation typically weakens multisensory body illusions and our data show that in this condition the participant experiences reduced enfacement for their unfiltered (current) face. We may speculate that a ‘dissociation’ from one’s adult body representation that occurs in the asynchronous condition may facilitate access to childhood episodic memories relative to the condition in which the adult body representation is stronger (synchronous condition), but further research would be necessary to test this idea^50^.

To conclude, the present study provides evidence of an interaction between bodily self and autobiographical episodic memory. We argue that the interaction occurs at the level of the construction of memory traces. However, an important limitation of the current research was that we did not observe an effect of visuo-motor synchrony on AM recollection. We recommend that future studies control for the resemblance of the morphed virtual face to the participant’s face. The current experiment did not assess the extent to which the participants felt that the morphed face resembled their childhood face. We recommend future laboratory-based studies measure the resemblance to one’s actual childhood face because variations in how similar the morphed face is to the childhood face could explain some of the variation in the effect of the illusion on different individuals. We also recommend a modified research protocol for future (lab-based) experiments in which the enfacement illusion questionnaire is completed after the AM interview and not before to avoid any expectancy effect or break in the impact of illusion on AM recollection. The study was further limited by not verifying the veracity of recollected memories. It is recommended to include measures of memory verification in future experimental designs to control for false or confabulated memories. It is also important to note that even though we achieved good to excellent interrater reliability for memory scoring, there was wide confidence interval for specific probing based memory scores. We also recommend future studies with more robust interrater reliability by achieving narrower confidence interval. Nevertheless, this study provides preliminary evidence of how temporary changes to the representation and experience of the bodily self may impact access to memory.

## Methods

### Participants

A total of 50 healthy adult (18–64 years) participants were included in this study. The mean age of the sample was 28.14 (*SD* = 4.94) years and there were 34 female participants and 16 males. The participants were randomly allocated into two groups (morphed: child face filter, unmorphed: no face filter). The groups were found to be adequately matched for age and gender. The mean age for ‘child face filter’ group was 28.28 (*SD* = 6.161) years and the mean age for ‘no face filter’ group was 28.00 (*SD* = 3.571) years. There was non-normal distribution of age for ‘child face filter’ group (*W* = 0.819, *p* <.001) so we conducted a Mann-Whitney U test to assess any differences in age between the groups. We found no significant difference in age for the two groups (*U* = 286.00, *p* =.605). We performed a chi squared test to assess the matching of gender for the two groups and found that the males and females were in the same proportions for the two groups (X^2^(1, *N* = 50) = 0.00, *p* = 1.000).

None of the participants had any self-declared history of previous psychiatric or neurological disorder. The experiment was conducted only if the participants had access to Wi-Fi with over 25 Mbps download speed, access to video conferencing software Zoom, had a laptop or personal computer with screen size more than 13 inches and with a webcam. Online informed consent was acquired by all the participants prior to participation. Informed consent was also acquired from the participants for their identifying image being used for the purpose of publishing in an online open-access publication. Ethical approval (approval number ETH2223-6198) was granted by the Faculty of Science and Engineering Research Ethics Panel at Anglia Ruskin University, Cambridge, UK. All experiments were performed in accordance with the relevant guidelines and regulations.

### Power calculation

A power calculation using G*power was computed to estimate the sample size for our study. Based on our primary research question, we planned to conduct a MANOVA with three response variables (averaged semantic memory scores, averaged free recollection based memory scores, and averaged specific probing based memory scores). Factors of group (child face filter, no face filter), visuo-motor synchrony (synchronous, asynchronous) and memory type (recent, childhood) were used. Using these parameters, we aimed to have a medium to large effect size (f = 0.35), α error probability of 0.05, and a power of 0.80. A medium to large effect size was selected for power calculation to ensure impactful findings were detected and to avoid overinterpretation of marginally significant results. A target of medium to large effect size (0.6) was also selected by Bréchet and colleagues investigating the impact of bodily self-consciousness on episodic memory^30^. Following the power calculation, we estimated our total sample size to be 48, and so we collected data from 50 participants. The sample size was also justified for the current study as it was comparable to previous literature investigating the impact of full body illusions on episodic memory^14,21,29^.

### Visual stimuli

**Fig. 3:**
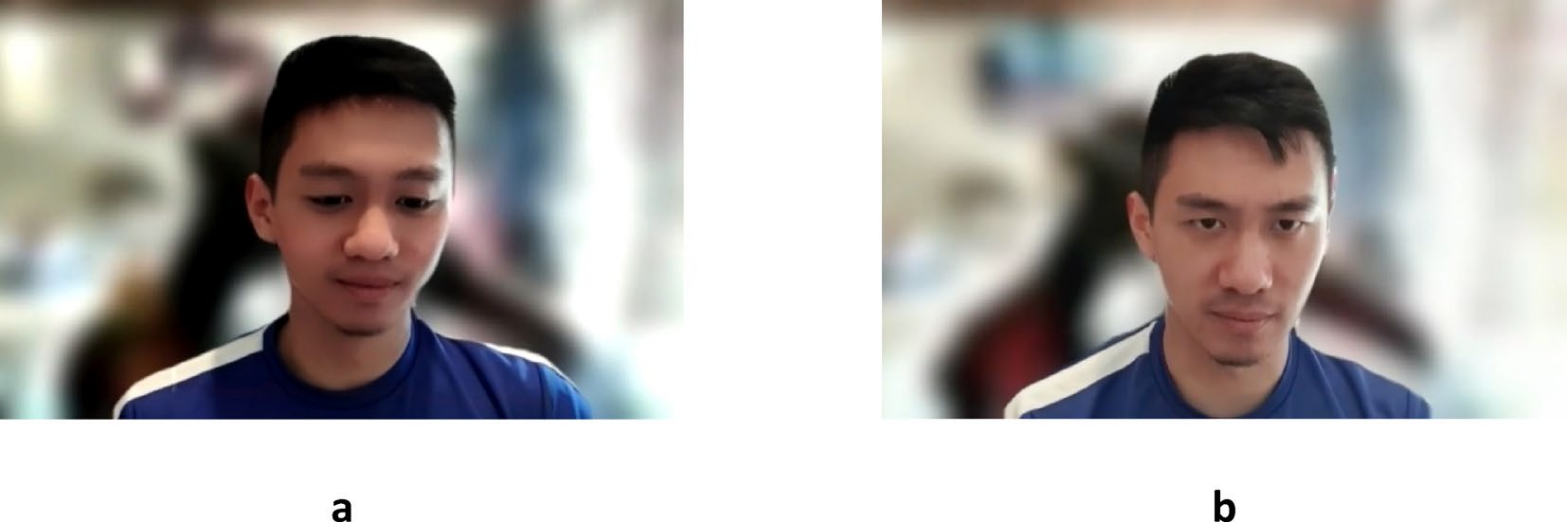
*Face stimuli used in the experiment. Note: ***a** – Child face created using ‘Baby’ filter of Snap Camera software, and **b** – No filter. Informed consent was acquired from the participant for their identifying image for the purpose of publishing in an online open-access publication.

In the ‘Child face filter’ group (*n* = 25), participants viewed a ‘younger version’ of their own face in a live video (see Fig. 3). This was created by using the ‘baby face’ filter of the Snap Camera software (Snap Camera, 2020). The other group (‘no face filter’ group; *n* = 25) viewed a live video of their face on the screen without any morphing or manipulation.

### Enfacement illusion

The enfacement illusion was created by manipulating the visuo-motor synchrony between the physical head movements of the participants and the video feed that the participants saw while making the head movements. In a practise phase before the main experiment, the participants were first shown a pre-recorded video of an actor moving their head with the repetitive motion of left-centre-right-centre around the vertical central axis at 45 bpm (beats per minute) for 90 s. The 45 bpm rate was timed and reinforced using a metronome. The participants were instructed to copy the head movement in order to practice. During the illusory manipulation, the participants looked at their own video feed while listening to 45 bpm metronome beats in the background. They were instructed to make similar head movements in left-centre-right-centre directions in time with the metronome beats, as they did during the practice session. This was again performed for 90 s. The duration was set at 90 s as previous literature have shown that 90 s are sufficient to successfully induce bodily illusion^51–53^. The video feed of participants faces was viewed via the self-viewing feature of the video conferencing software Zoom^54^. To create either a strong or weak enfacement illusion, the mirroring of the participant’s video feed was manipulated. In the strong (synchronous) enfacement illusion condition, the participants viewed a mirrored video feed in which the face on the screen moved in the same direction as they moved their head. In the weak (asynchronous) enfacement illusion condition the participants viewed an unmirrored video feed in which they saw their face on the screen move in the opposite direction to which they moved their head.

### Enfacement illusion questionnaire

Susceptibility to the illusion was measured using a modified enfacement illusion questionnaire based on the modification of a standardised avatar embodiment questionnaire for body illusions as reported by Peck and Gonzalez-Franco^55,56^. The modification in the questionnaire for enfacement of a virtual face was based on a questionnaire used in previous enfacement illusion study by Serino and colleagues^34^. The questionnaire was a 12-item questionnaire that was completed by the participants immediately after each synchronous or asynchronous visuo-motor manipulation. The questionnaire was displayed on the screen by replacing the video feed and the participants responded by using their computer mouse or touchpad. Each questionnaire item was scored on a 7-point Likert scale, from strongly disagree to strongly agree. The subjective experience of face ownership was measure by items 1, 2, 3 and 4; face agency was measured by items 5, 7 and 8; the feeling of being younger by items 9 and 11; the feeling of being an adult by items 10 and 12; and there was a control question (item 6); see Table 4. The participants were also given an option to type about their subjective experience with a maximum limit of 100 words.

**Table 4:** Modified enfacement illusion questionnaire with 12 items.

Item	Statement*
**1**	… It seemed like I was looking directly at my own face, like in a mirror.
**2**	… it seemed like the virtual face began to resemble my real face.
**3**	… it seemed like the virtual face was my face.
**4**	… It seemed like my own face became virtual.
**5**	… It seemed like I could have moved parts of the virtual face if I wanted to
**6**	… It seemed like I could have more than one face.
**7**	… It seemed like I was in control of the virtual face.
**8**	… It seemed like I was the cause of Virtual face movements.
**9**	… It seemed like I was a child.
**10**	… It seemed like I was an adult.
**11**	… It seemed like I was of younger age than I actually am.
**12**	… It seemed like I was of the same age that I actually am.

Note. Star (*) – Each statement in the digital version of the questionnaire started with ‘During the experiment, there were times when …’. The illusion questionnaire is a modified version of the questionnaire used in a previous study^34^.

### Autobiographical memory interview

A modified autobiographical memory interview (AMI) was conducted^38,57^. After the participants completed the enfacement illusion questionnaire, they were once again shown their morphed or unmorphed face on the screen depending on the group they were assigned to. While they continued to view their morphed or unmorphed face along with their assigned video feed manipulation (mirrored or unmirrored), they were asked a set of questions about their autobiographical memories. However, the participants were not instructed to perform any specific head movements during the AMI. The following instruction was given to the participants prior to each memory recollection^38^ :

“I am going to ask you to tell me about an event from two time periods of your life. It can be either your childhood memory comprising of events that took place during your early childhood until the age of 11 years, or a recent memory which comprises of events that happened in previous one year. You will be given cue words ‘Home’ or ‘Holiday’ based on which you can choose any related event you wish. I will ask you to describe the events for a maximum of 5 minutes. I will then ask some questions about them to acquire further details, so be sure to only choose events that you feel comfortable discussing in detail. The event must be one you were personally involved in, and you must have a recollection of being personally involved. Do not pick events that you have heard about from others. They must be detailed events from a specific time and place. For example, simple recollection of having played a basketball game would not be sufficient. However, an event involving a specific basketball game would be good. I want you to provide as much detail as you can about the event. Our interest is not in the specific characteristics of the event you choose, but rather the amount of detail you can remember about that event. So, I would again advise you to only pick events that you are comfortable sharing with me.”

The AMI consists of 4 broad components – semantic recollection, free recollection, recollection after general probing, and recollection after specific probing^38^. However, in the experiment by Levine, there was no significant difference in the scoring for free recollection and general probing. Therefore, in the current study, the memories recollected during these phases were scored together and are categorised as just the free recollection based episodic memory scores^38^. As a result, the AMI scoring in the current study has been spilt into 3 sections: (i) semantic, (ii) free recollection, and (iii) specific probing based memory scores. The participants were asked to recollect either a childhood memory or a recent memory based on the cue words ‘home’ or ‘holiday’. There were three initial semantic (factual) questions asked. If the memory to be recollected was for childhood holiday visit, the following was asked:

- Can you remember the name of a place you have visited when you were 11 years old or younger?
- Can you remember the year when the visit took place?
- Can you remember the full name or just the first name of a person who accompanied you, or that of a person you met during the visit?

If the memory to be recollected was for childhood home, the following was asked:

- Can you remember the name of the place where you have lived for the longest duration when you were 11 years old or younger?
- Can you remember the duration in years when you lived in that home?
- Can you remember the full name or just the first name of a neighbour or a friend when you lived there?

For recent home and holiday memories, the same questions were asked, but limited to only events from previous one year.

After the semantic questions, the participants were asked to freely recollect an episodic autobiographical memory of an event for 3–5 min. Following the free recollection, the participants were probed using a general prompt: ‘Is that everything you can recall about this incident?’. During this general probing phase, the interviewer also guided the participant to recollect just one event if they recollected multiple events during the free recollection. This was followed by specific probing phase during which the interviewer asked specific pre-structured questions to facilitate detailed recollection of event related episodic information related to time, place, episodic richness, sensory perceptions, and thoughts and emotions. Examples of specific probing:

- Can you further recall what happened during that [main event]. For example, who else was there? What happened immediately before or afterwards? What was the weather like? How did you feel at the time? What was the behaviour or reactions of others around you?
- Can you recall in more detail the day, week or season, or the time of day at which the event (or parts of that event) occurred?
- Can you further recall the details of the location of the [main event] for example, the town or city, street, building, room, or the outdoor location?
- Can you further recall the details of memory regarding what you heard, smelled, touched, tasted, saw, your body position (e.g.: were you sitting, lying down, standing etc.,) and what was the duration of the [main event]?
- Can you recall your emotional state and thoughts during the [main event]?

### Autobiographical memory interview scoring

Scoring of the AMI was split into 3 sections based on the protocol used by Levine and colleagues^38^. The first section of scoring was for semantic questions. Successful recollection for each semantic question was awarded 1 point. If the participant recalled just the first name in the third semantic question, they were awarded 0.5 point for that question. Thus, each participant could score a maximum of 3 points for this section. The composite semantic scores for further analyses were calculated as the average of scores for the three semantic questions resulting in values ranging between 0 and 1. The next section of scoring was for free recollection. Information was extracted based on the following six criteria – episodic richness, time, place, sensory perceptions, thoughts and emotions, and time integration. Episodic richness related to the qualitative estimate of reexperiencing. Time was scored based on the information recollected as it related to year, season, month, day of week and time of day. Place was scored for localisation of an event including, e.g., the city, street, building, room, and parts of room. Perceptual scores were based on auditory, olfactory, tactile, taste, visual details, body position and duration of the event. Scores for thoughts and emotions were given based on the emotional state and thoughts during the event. Time integration score was given for the event’s integration into a larger time scale as evidenced by inclusion of temporal contextual information or relation to other life periods. These six criteria were separately scored on a 4-point scale (0–3) as follows^38^:

*3 points* – A rich, highly specific, evocative, and/or vivid description that appears to emerge from a feeling of reexperiencing.

*2 points* – A detailed description that falls short of 3 points in the degree of richness.

*1 point* – A description that is limited to general, nonspecific information but still episodic in nature.

*0 points* – No mention of information pertaining to the specified category, or a response that is based on semantic knowledge rather than episodic memory.

The third section concerned memory details recollected during the specific probing phase. Similar to the previously described section, this section was also scored on separate 4-point (0–3) scales for the 6 criteria – episodic richness, time, place, sensory perceptions, thoughts and emotions, and time integration. Composite free recollection based memory scores and composite specific probing based memory scores were calculated as the average of scores for the six criteria resulting in values ranging between 0 and 3.

### Experimental design and procedure

The online experiment was created using the software Gorilla experiment builder^58^. The researcher was in contact with the participant during the experiment by means of the video conferencing software Zoom^54^. Participants were provided with a link that opened the Gorilla platform on their browser^58^. On the platform they read through the information sheet and provided informed consent before starting the experiment. They were then directed to a zoom call in which the researcher was already present. Following this, they were instructed to make the practice head movements for 90 s. They were then randomly assigned to the child face group or no filter group. This randomisation was counterbalanced to have 25 participants assigned to the child face group and the remaining 25 to the no filter group. Every participant participated in four experimental conditions based on the combinations of visuo-motor synchrony (synchronous, asynchronous) during the enfacement illusion and type of memory probed (recent, childhood). The conditions were (i) synchronous-recent, (ii) asynchronous-recent, (iii) synchronous-childhood, and (iv) asynchronous-childhood. The four experimental conditions were in a randomised and counterbalanced sequence. In each condition, the participants were instructed to make the head movement (synchronous or asynchronous) for 90 s while watching the video feed of their own face. Immediately after the head movements, the participants completed the enfacement illusion questionnaire. Immediately after that the AMI was conducted, and the interview was recorded for it to be later scored and analysed. After the completion of the four experimental conditions, the participants were debriefed, and the experiment concluded.

### Data analysis

Data collection from the four experiment conditions per participant resulted in a total of 200 memory interviews (i.e., four memories per participant). The scoring procedure and criteria were first discussed by UG, PB, and PRP. Following that, memory interviews from 10 randomly selected participants were scored separately by UG and PRP. The raters were blind to the group distribution of the participants. Inter-rater reliability was assessed using separate interclass correlation coefficient (ICC) test for composite (average) scores of the 3 sections of AMI – (i) semantic scores; (ii) scores for free recollection; and (iii) scores for specific probing. ICC estimates and their 95% confident intervals were calculated based on a mean-rating (*k* = 2), absolute-agreement, 2-way mixed-effects model. Acceptable ICC test results were achieved (values over 0.75) after two attempts of scoring memory interviews from 10 randomly selected participants for each try. The interviews from the remaining 40 participants were scored by the two raters (20 participants each by UG and PRP).

Data collection from the four experiment conditions per participant resulted in a total of 200 memory interviews. The statistical analysis for this experiment were performed using SPSS statistical package version 28.0.0.0 (SPSS Inc, Chicago, IL). Descriptive statistics were calculated, a mixed model 2 × 2 × 5 MANOVA was conducted to validate our illusory manipulation, and a mixed model 2 × 2 × 2 × 3 MANCOVA with age as a covariate was conducted to test our hypotheses.

The 2 × 2 × 5 mixed model MANOVA was conducted to examine the effect of between-subject factor group (child face filter, no face filter) and within-subject factor visuo-motor synchrony (synchronous, asynchronous) on enfacement scores - face ownership, face agency, child-like experience, adult-like experience, and control scores. The four within-subject sessions of visuo-motor synchrony had two synchronous sessions and two asynchronous sessions. The enfacement scores from two synchronous sessions and two asynchronous sessions were averaged separately and analysed. The 2 × 2 × 2 × 3 mixed model MANCOVA was performed to analyse the effect of between-subject factor group (child face filter, no face filter), within-subject factor visuo-motor synchrony (synchronous, asynchronous), and within-subject factor of AM life period (recent, childhood) on AM (200 memories) recollection scores – (i) semantic, (ii) free recollection, and (iii) specific probing based memory scores controlling for age as a covariate. Assumptions of linearity and homogeneity of regression slopes were checked for age as a covariate. Linearity was tested by visually inspecting for linear patterns in scatterplots between age and dependent variables grouped for each independent variable condition. Homogeneity of regression slopes were tested by examining interactions between age and independent variables. Univariate outliers were identified using boxplots. For multivariate statistical tests, normality and sphericity were checked for any violation of assumptions using Shapiro-Wilk and Mauchly’s test. Multivariate analyses were additionally checked for multicollinearity. Univariate and multivariate outliers were identified using box plots and by calculating the Mahalanobis distance and excluded from the analyses. Pairwise comparisons were conducted with Bonferroni adjustment. An alpha level of 0.05 was implemented.

Correlations were run to test for any associations between subjective enfacement experience (face ownership scores and face agency scores) and AM performance (composite semantic AM scores, composite episodic memory scores based on free recollection, and composite episodic memory scores based on specific probing). Correlations were run separately for childhood AM and recent AM for different conditions of visuo-motor synchrony (synchronous, asynchronous) and group (child face filter, no face filter). The normal distribution of variables and presence or absence of outliers were checked for any violations of correlation assumptions. Depending on the presence or absence of any violation of assumptions, parametric or non-parametric correlation tests were performed. Holm-Bonferroni correction was applied to reduce type I error for multiple comparisons. There was a total of 48 comparisons, resulting in an adjusted significance level of 0.001 instead of 0.05.

## Data Availability

The data that support the findings of this study are available in Open Science Framework (OSF) at https://doi.org/10.17605/OSF.IO/6QF3M.
